# Predictors of acute stress disorder following a military maritime accident

**DOI:** 10.3389/fpsyt.2025.1654552

**Published:** 2025-10-23

**Authors:** Sverre Sanden, Jarle Eid, Sigurd William Hystad

**Affiliations:** ^1^ Centre for Crisis Psychology, University of Bergen, Bergen, Norway; ^2^ Royal Norwegian Navy Medical Services, Norwegian Armed Forces, Oslo, Norway; ^3^ Department of Psychosocial Science, University of Bergen, Bergen, Norway

**Keywords:** acute stress (disorder), screening, maritime, navy, military personnel, accident & emergency

## Abstract

The present study examines the prevalence and predictors of symptoms of acute stress disorder (ASD) in crew members of Norwegian frigate *HNoMS Helge Ingstad* (n = 118) following the November 8^th,^ 2018, collision with civilian oil tanker *Sola TS*, which led to grounding and total loss of the frigate. Collected six months prior to the accident (T1), pre-deployment scores on the General Health Questionnaire (GHQ-12), Hopkins Symptom Checklist 25 (HSCL-25) depression items, HSCL-25 anxiety items and professional self-efficacy were examined as predictors of scores on the Acute Stress Disorder Scale (ASDS) measured three weeks post-accident (T2), along with sex, personnel category, operational experience, and peri-traumatic perceived control and perceived coping, also collected at T2. Results show 28% of participants obtained scores indicating clinically significant symptoms of ASD. Baseline HSCL-25 anxiety, HSCL-25 depression and female sex were positively related to ASDS scores. Perceived control in the situation was negatively related to ASDS scores. Other factors were not predictive. Findings demonstrate that even slight elevations in pre-incident scores on symptoms of anxiety and depression increase risk for significant symptoms of ASD in military populations and suggest pre-deployment screening could help identify subgroups at higher risk of developing ASD after maritime accidents.

## Introduction

Operational personnel, such as soldiers, sailors, police officers, and firefighters, are at risk of experiencing potentially traumatic events (PTEs). While the most common developmental trajectories following PTEs are resilience and rapid recovery, a significant minority of those exposed develop debilitating symptoms of acute and post-traumatic stress (1–3). Operational personnel usually experience PTEs while working in dyads or small teams – the most common work groups for operational personnel – but larger-scale PTEs involving entire units, crews or organizations also occur. Notable examples of the latter, include the collision of the USS *Fitzgerald* (4) and the Grenfell Tower fire in London (5). Major PTEs involving large groups of personnel often trigger group-level psychosocial interventions, with the early prevention of acute and post-traumatic stress as primary objectives (6). Interventions occurring after exposure to a PTE but before the onset of significant symptoms of any duration are referred to as “indicated prevention” (7). However, research on the effectiveness of indicated preventive efforts in reducing later symptoms is sobering. Recent reviews indicate limited effectiveness of early prevention of post-traumatic stress, with stronger support for individual treatment of those who later report significant symptoms (8–10).

One of the challenges in group-based indicated preventive efforts is the heterogeneity of reactions to PTEs and the difficulty in predicting who will develop significant symptoms of acute and post-traumatic stress (9, 11). As noted in a recent systematic review and meta-analysis, reported rates of acute stress disorder (ASD) vary widely across studies and seem to depend on demographic variables, type of incident, geographic location and the assessment instrument used (12).

Understanding the factors that predispose operational personnel to severe ASD symptoms is crucial for enabling targeted early interventions for high-risk individuals. This helps target support to those who need it most and avoids unnecessary measures for those who are more resilient. To do this, we need better knowledge and tools to predict who is at risk.

Extensive research has explored the prediction and prevention of post-traumatic stress reactions, examining a range of protective and risk factors (8, 9, 11). Temporally, these factors are classified as pre-exposure, peri-traumatic, and post-exposure. *Pre-exposure factors* are individual differences present before a PTE occurs and include sex, personality dimensions, history of mental health problems and previous traumatic experiences. *Peri-traumatic factors* occur during a PTE and include elements such as intensity, duration, and subjective perceptions of threat. *Post-exposure factors* take effect after a PTE and include elements such as perceived social support and coping efficacy, which refers to victim’s perceived ability to cope with their own reactions.

Results from recent studies indicate that a variety of measures, including some very brief, can be used to predict the risk of severe post-traumatic stress. These measures exploit various pre-exposure factors, medical history, biological data, and brief screening instruments (13–15). For example, a study on US Army personnel deploying to a warzone used pre-deployment polygenic, epigenetic, metabolomic, endocrine, inflammatory and routine clinical lab markers, computerized neurocognitive testing and self-reported psychological symptoms to predict post-traumatic stress disorder (PTSD) post-deployment (16). The proposed model achieved high discriminatory power, with pre-deployment sleep quality, anxiety, depression, sustained attention and cognitive flexibility to be the highest ranked predictors, and blood-based biomarkers complementing the most important predictors.

Prospective studies on ASD are quite rare, likely due to the methodical challenges of measuring predictive factors before a PTE occurs. However, some civilian organizations (17) and most military services routinely perform health screenings and gather pre-deployment data on physical and mental fitness, data which could inform prospective studies.

The Royal Norwegian Navy (RNoN) has established routines for personnel care before, during and after deployments to international operations and following PTEs (Sanden et al., 2014; 2024). These routines involve the use of screening questionnaires, containing validated measures of psychological symptoms and questions composed by practitioners to assess significant dimensions of deployment and personal and organizational functioning such as motivation, well-being, and family support (18).

In May 2018, crew members of the RNoN frigate *His Norwegian Majesty’s Ship* (*HNoMS*) *Helge Ingstad* filled out screening questionnaires as part of their pre-deployment routines before deploying to Standing NATO Maritime Group No. 1 (SNMG1). Screening is in part done to identify crew members not fit for deployment and thus functions as a form of selection. Crew members with elevated symptom scores are contacted by clinicians for follow up, and those considered not fit for duty are excluded from deployment. On November 8^th^, 2018, at 04:01 a.m., *HNoMS Helge Ingstad* collided with a civilian oil tanker in the coastal waters outside Bergen, Norway. The tanker struck the frigate with the side of its bow, pushing it, while the tanker’s anchor tore a 46-meter (150 foot) gash down the starboard side of the frigate. Several berthing compartments were crushed, others immediately flooded with seawater, and the frigate suffered a blackout and temporary loss of steering. To prevent further flooding, watertight compartments were sealed off before all crew members were accounted for. Several crew members had to climb through the gash in the starboard hull to escape from their berthing compartments and move to a higher deck.

**Figure 1:**
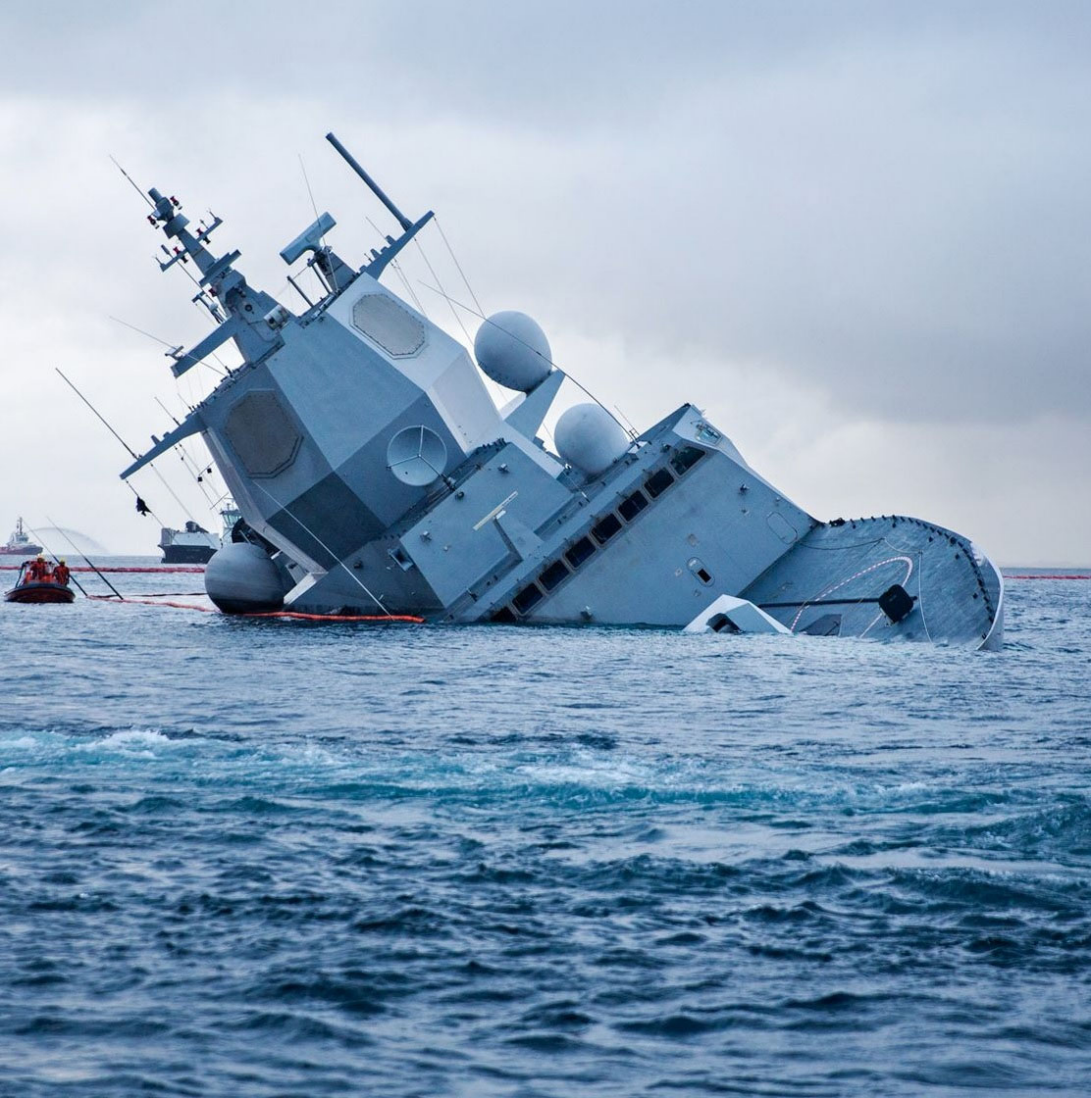
HNoMS Helge Ingstad grounded after colliding with civilian oil tanker. Source Norwegian Armed Forces Media Bank, https://www.forsvaret.no/aktuelt-og-presse/mediebank.

Although no crew members were killed or seriously injured, the severity and suddenness of the incident justify its classification as a PTE. The accident occurred at night under unclear circumstances, involving all crew members in a life-threatening situation. Several individuals narrowly escaped being crushed or swept overboard and had to fight for their survival. Others worked intensely to save the ship and their comrades, knowing that fellow crew members were unaccounted for and believing they were either trapped in flooded compartments or already dead. Despite intense efforts to control the damage and the ship, the accident led to grounding, evacuation, and total loss of the frigate [see **Figure 1**; for a full description of the accident, see report from Accident Investigation Board Norway (19)].

Following evacuation, crew members were transported to an improvised reception center at a nearby naval base and began a structured program of group-based early psychosocial intervention and return-to-work procedures. The program included psychological first aid, psychoeducation, group sessions, individual counselling, and exposure training. As part of the exposure training, the crew boarded an identical frigate and underwent training to return to sea (6). All crew members participated in the psychosocial intervention, which was completed two weeks after the accident. Three weeks after the accident, crew members filled out screening questionnaires as part of Navy routines following a PTE.

The incident provides an opportunity to investigate the occurrence of ASD in a particular sample and setting: a selected and trained military crew involved in a serious peacetime accident and subject to a comprehensive, structured preventive psychosocial intervention. It also provides an opportunity for investigating predictors of ASD assessed six months pre-accident. While several studies have identified individual and contextual factors associated with post-traumatic stress over time, few have investigated prospective factors associated with ASD.

The current study will investigate the occurrence of ASD symptoms and whether pre-exposure and peritraumatic factors can help explain variations in symptoms. Results could contribute to improved prediction of symptom development following PTEs and have implications for the design of indicated preventive efforts, potentially leading to more targeted interventions. Additionally, findings could increase knowledge about the occurrence of ASD symptoms despite extensive early preventive efforts, highlighting the need for further psychological treatment. Studies on comparable populations and incidents have found that around 50% of exposed personnel express a need for mental health support during the first year following maritime accidents (20). Additionally, news reports have highlighted the severe consequences military maritime accidents can have for later operational readiness and work capacity (4, 21). Early identification of individuals at risk could facilitate more timely contact with therapists and shorter courses of treatment.

Our main research aim was to investigate potential pre-exposure and peritraumatic predictors of ASD. Pre-exposure levels of psychological symptoms have been linked to the likelihood of experiencing symptoms of post-traumatic stress following critical incidents, with higher symptom levels pre-incident indicating a greater likelihood of developing more severe post-traumatic symptoms (16, 22–24). We presume the same to be true for ASD and accordingly hypothesize that:

H1: Higher level of general psychological distress prior to the accident is related to more symptoms of ASD following a naval accident.

H2: Higher level of anxiety symptoms prior to the accident is related to more symptoms of ASD following a naval accident.

H3: Higher level of depression symptoms prior to the accident is related to more symptoms of ASD following a naval accident.

Compared with men, women have been found to have a twofold risk of developing post-traumatic stress disorder after exposure to trauma (25). Additionally, being of the female sex has been associated with a higher likelihood of developing symptoms following potentially traumatic military maritime incidents (26). Based on this, we hypothesized that:

H4: Female crew members report more symptoms of ASD than male crew members.

Self-efficacy has been associated with lower levels of acute and post-traumatic symptoms, indicating a role in predicting post-trauma resilience (27, 28). Accordingly, we hypothesized that:

H5: Higher level of professional self-efficacy prior to the accident is related to less symptoms of ASD.

Perceived control and perceived coping in the face of adversity have been conceptually and empirically linked to reduced negative psychological impact following PTEs (28–30). The perceived lack of coping during maritime accidents has previously been identified as a vulnerability factor for post-traumatic symptoms (31). We therefore propose perceived control and perceived coping to be peritraumatic factors that can help explain variations in symptoms of ASD:

H6: Higher level of perceived control during an accident is related to less symptoms of ASD.

H7: Higher level of perceived coping during an accident is related to less symptoms of ASD.

Building on the concepts of perceived control and coping, we hypothesize that individuals with greater operational experience are likely to be better mentally prepared and possess more effective coping resources during a critical incident. We therefore hypothesized that:

H8: More operational experience is related to less symptoms of ASD.

Finally, the personnel category to which participants belong (conscript, officer, other ranks, or apprentice) is related to factors such as selection, social role, position on the ship, level of training, and level of experience. Previous studies on symptoms of post-traumatic stress following maritime accidents and attacks have shown that contextual factors, such as social role, are strongly associated with subsequent stress reactions, with conscripts being more likely to experience higher levels of post-traumatic stress (26, 32, 33). Apprentices are of similar age and rank as conscripts. We therefore hypothesized that:

H9: Apprentices and conscripts experience more symptoms of ASD than other personnel categories.

## Materials and methods

Pre-deployment and post-accident data were collected for operational and clinical purposes by a team of clinical psychologists employed by RNoN Medical Services as part of naval routines and procedures. More than two years after the accident, crew members were contacted and informed about the current research project. After obtaining consent from participants, data were transferred to the Norwegian Armed Forces Health Registry (NAFHR), which controls research access to health data involving Norwegian Armed Forces personnel. While the screening followed established routines, the transfer of questionnaire data to NAFHR was based on informed consent, as data transfer opens for questionnaire data to be shared for research purposes upon application. The current study was approved by the Regional Committee for Medical and Health Research Ethics for Western Norway (case number 283027) and access to data was granted by NAFHR in September 2021. Before sharing, NAFHR pseudonymized data and recoded certain variables into categories to prevent indirect identification of participants.

### Participants and procedure

All participants had been screened for general mental abilities upon entering the armed forces, ensuring that they had mental abilities within the normal range. Participants were not screened for socioeconomic status or educational level. Conscripts and apprentices assigned to ship duty must achieve top score on their mental health evaluation upon enrolment in the Norwegian Armed Forces. While this score is primarily based on self-reporting and may be influenced by underreporting, it indicates that apprentices and conscript participants did not have a history of mental illness or stress-induced psychological disorders. Officers and other ranks go through regular medical fitness-for-duty evaluations, including the assessment of mental health, to maintain their certified fitness for ship duty.

Data were collected at two timepoints, T1 and T2. Data collection at T1 took place six months prior to the accident, as part of a routine pre-deployment screening. Paper questionnaires were handed out by RNoN clinical psychologists, completed by crew members, and collected afterwards. Of the 137 crew members aboard the ship at the time of the accident, 62 crew members (45%) had completed the routine pre-deployment screening and consented to have their data transferred to NAFHR.

Data collection at T2 was conducted three weeks after the accident, as part of routine personnel care following critical incidents in the RNoN. Paper questionnaires were distributed by RNoN clinical psychologists, completed by crew members, and collected afterward. Of the 137 crew members aboard the ship at the time of the accident, 118 (86%) consented to having data from this screening transferred to NAFHR.

There were several reasons for the limited overlap in participants at T1 and T2. First, there is a constant rotation of personnel in navy crews, as conscripts are discharged and new conscripts are recruited, and as officers and other ranks regularly change positions or crews. Second, pre-deployment screening was conducted aboard the ship at a time of convenience before deployment, and some crew members were not present to complete screening. Third, the accident happened during transit after the crew had participated in a major exercise lasting several weeks, and some crew members had left the ship for leave after the exercise. Fourth, some of the personnel onboard at the time of the accident were not regular crew members, but substitutes, instructors, or similar personnel, and had thus not completed pre-deployment screening.

Demographic information for samples at T1 and T2 is presented in **Table 1**. Participants at T1 (n = 62) included 13 females (21%) and were composed of 26% apprentices and conscripts, 29% officers, and 45% other ranks. Participants at T2 (n = 118) included 18 females (15%) and were composed of 32% apprentices and conscripts, 22% officers, and 46% other ranks. Due to concerns about possible indirect identification, the age of female participants was not categorized by NAFHR. Thus, the age information in **Table 1** applies only to male participants. Similar concerns of possible indirect identification led to the categorization of length of service on the Nansen-class frigate into just two categories at T1 (≤ 35 months and ≥ 36 months). The composition of personnel categories and participant demographics at T2 were representative of frigate crews in the RNoN (author correspondence with RNoN staff, May 2023).

**Table 1:** Demographic information for participants at time 1 and time 2.

Demographic	Time 1 *N* = 62		Time 2 *N* = 118	
	No.	%	No.	%
Gender				
Male	49	79	100	85
Female	13	21	18	15
Age group^a^				
≤ 20 years	6	12	19	19
21–25 years	18	37	31	31
26–30 years	15	31	28	28
31–60 years	10	20	22	22
Personnel class				
Apprentices and conscripts (OR1-OR2)	16	26	38	32
Officers (OF1-OF4)	18	29	26	22
Other (OR3-OR7)	28	45	54	46
Length of service^b^				
< 12 mos.	21	34	34	29
12–35 mos.			40	34
≥ 36 mos.	41	66	44	37

The ranks OR1 and OR2 include Ordinary Rating and Able Rating, as well as enlisted Seaman. The ranks OF1 to OF4 include Sub Lieutenant, Lieutenant, Lieutenant Commander, Commander, and Commander Senior Grade. The ranks OR3-OR7 include Leading Seaman, Master Seaman, Petty Officer, Petty Officer 1^st^ Class, Senior Petty Officer, and Chief Petty Officer.

a Due to issues of potential indirect identification, the ages of female participants were not categorized by the Norwegian Armed Forces Health Registry (NAFHR). Reported age groups and percentages thus apply to males only.

b Due to issues of potential indirect identification of participants, the length of service was categorized into only two groups at T1.

The difference in the distribution of time of service at T1 and T2 illustrates that turnover among less experienced personnel was a major cause for the limited overlap in participants. A quarter of conscript crew members are rotated every three months, as they complete a 12-month military service, with new cohorts being recruited and dismissed every three months. Only 21 participants with less than 36 months of service at T1 were also present during the accident six months later. The total number of participants at T2 with less than 36 months of service was 74, meaning that 53 participants with less than 36 months of service had not completed screening at T1, giving in an overlap of participants from T1 to T2 of 28.4%. For participants with more than 36 months of service the overlap is much higher, with 41 out of 44 participants (93.2%) who were present at the accident also having completed the pre-deployment screening.

### Measures

#### Time 1

##### General health questionnaire

The 12-item General Health Questionnaire (GHQ-12) is a self-report screening measure for psychological distress. Items are scored on a four-point Likert type scale, ranging from absence of experienced distress or even perceived improved functioning (0) to a high degree of experienced distress (3). GHQ-12 has previously been used in both civilian and military populations (34, 35). In the current study, the mean score of all items was used to indicate the level of pre-exposure psychological distress.

Cronbach’s alpha for GHQ-12 was 0.64, which suggests potentially problematic internal consistency. Although originally proposed and typically used as a unidimensional measure, there is some debate regarding the dimensionality of the GHQ-12. A common finding from many studies is the lack of support for a single-factor structure, instead finding support for either a two-factor or three-factor structure. Complicating matters further is the concern that the division of GHQ-12 into positively and negatively phrased items may introduce method effects that artificially split it into separate factors (36). We acknowledge the low reliability in our study, which may partly be due to some multidimensionality present in the measure, but we nevertheless chose to keep it as a single index, as is most common in the literature.

##### Hopkins Symptom Checklist 25

Symptoms of anxiety and depression were measured using the Norwegian version of the Hopkins Symptom Checklist 25 (HSCL-25), a shortened version of the Symptom Checklist (37). The HSCL-25 is a 25-item self-report measure assessing symptoms of anxiety and depression experienced during the past two weeks (38) It uses a 4-point Likert scale with the following response options: *not at all* (1), *a little* (2), *quite a bit* (3) and *extremely* (4).

For the anxiety items (1-10), Cronbach’s alpha in the sample was 0.81, and for the depression items (11-25) it was 0.83, indicating good internal consistency for both scales. For the statistical tests, we calculated the mean scores for the anxiety items and the depression items.

##### Professional self-efficacy

Perceived self-efficacy is the personal belief in one´s capability to produce given attainments (39). As self-efficacy is domain specific, “*scales of perceived self-efficacy must be tailored to the particular domain of functioning that is the object of interest”* (Bandura, 2006, p. 307) (40). The current study was interested in how crew members handle a critical incident on a warship and relevant measures of self-efficacy should thus target crew members’ beliefs in their professional abilities as sailors and their perceived level of preparedness and skill.

At T1, personal sense of preparedness was assessed using the item “I feel well prepared for the mission ahead”, scored on a five-point Likert-type scale ranging from *not true* (1) to *absolutely true* (5). Perceived ability to solve the current mission was measured by the item, “In total, my perceived ability to solve the mission is: “, scored on a Likert-type scale ranging from *very poor* (1) to *very good* (5). The scores from these two items were then averaged to create a pre-incident self-efficacy index.

#### Time 2

##### Acute stress disorder scale

Symptoms of ASD were measured using the Norwegian version of the Acute Stress Disorder Scale (ASDS; Bryant et al., 2000). The ASDS is a self-report measure consisting of 19 items, divided into four subscales: five dissociative items, four re-experiencing items, four avoidance items, and six arousal items. All items are scored on a five-point Likert scale ranging from *not at all* (1) to *very much* (5), giving a total score and scores on the four subscales.

Bryant and colleagues (41) investigated the usefulness of the ASDS in identifying cases of diagnosable ASD according to DSM-IV criteria. Their results indicated that a score on the dissociation items ≥ 9, combined with a cumulative score ≥ 28 on the remaining items, was the most useful for correctly identifying cases of ASD. These suggested sum scores are equivalent to a mean score ≥ 1.8 on dissociation (i.e., a score of 9 divided by 5 dissociation items) and a mean score ≥ 2 (28/14) on the remaining ASDS items. As the DSM-IV is no longer the current diagnostic standard, we see these suggested cut-off scores as indicators of clinically significant symptoms of ASD rather than indicators of diagnosis.

Cronbach’s alpha for the ASDS in our sample was 0.89, indicating good internal consistency. For the subscales, Cronbach’s alpha values were as follows: dissociation = 0.73, re-experiencing = 0.68, avoidance = 0.61, and arousal = 0.77. These values suggest internal consistency ranging from potentially problematic to acceptable across the subscales.

ASDS subscales have shown variable internal consistency across various populations and settings (42–44), ranging from .70 to .93. The reasons for these variations remain unknown. In our case, it is possible that the psychosocial intervention mentioned may have affected the internal consistency of the subscales, particularly for the avoidance items. For example, one avoidance item addresses trying not to think about the incident, while another item addresses trying to avoid situations or people who remind you of the incident. While trying to avoid disturbing thoughts is possible, it is hard to avoid situations or people who remind you of the incident while being part of a collective psychosocial intervention specifically designed to promote exposure to anxiety-triggering stimuli (6). This could potentially influence internal consistency of the subscale. While acknowledging the moderate internal consistency, we chose to maintain the use of ASDS subscales for analysis.

##### Perceived control and perceived coping

Perceived control during the accident was measured by the item: “I felt that I was in control of the situation during the incident”. Perceived coping was measured by the item “I feel that I coped with the incident in a good way”. Both items were scored on a five-point Likert-type scale ranging from *not true* (1) to *absolutely true* (5).

##### Operational experience: time of service on Nansen-class frigate

Operational experience was measured by crew members writing down their total years and months of service on Nansen-class frigates. Reported scores were later transformed into number of months.

### Statistical analyses

To test our hypotheses, we conducted two series of multiple regression analyses. In the first series, ASDS and the four subscales were regressed on our pre-exposure variables psychological distress, anxiety, depression and self-efficacy. These analyses were conducted on the subsample that had completed the pre-deployment screening and were aboard the ship at the time of accident (*n* = 62).

The next series of multiple regressions regressed ASDS and the subscales on the peritraumatic variables perceived control and coping during the incident, as well as on operational experience, sex and personnel class. These analyses were conducted on the sample of crew members aboard the ship at the time of the accident who gave their informed consent (*n* = 118). All analyses were conducted using Stata 18.5 (StataCorp, 2024).

### Use of generative AI

Generative AI was used in the preparation of this manuscript for the purposes of language editing, grammar checking, and linguistic enhancement. The tool employed was the University of Bergen’s internal version of Copilot, powered by the large language model GPT-4 developed by OpenAI.

## Results

**Table 2** presents mean scores, standard deviations, reliability coefficients, and correlations between all variables included in this study.

**Table 2:** Descriptive statistics, reliability coefficients, and inter-correlations between all variables.

Variables	*M* (*SD*)	1.	2.	3.	4.	5.	6.	7.	8.	9.	10.	11.	12.
Time 1 (n = 62)													
1. HSCL-25 depression	1.19 (0.22)	**.83**											
2. HSCL-25 anxiety	1.16 (0.17)	.58***	**.81**										
3. GHQ-12	1.69 (0.20)	.55***	.41***	**.64**									
4. Self-efficacy	3.98 (0.80)	-.18	-.26*	-.08	**.27** ^a^								
Time 2 (n = 118)													
5. Sex	.85^b^	-.11	-.10	.09	.08	—							
6. ASDS total	1.91 (0.60)	.46***	.46***	.24	-.16	-.24**	**.89**						
7. ASDS dissociation	2.21 (0.81)	.50***	.47***	.25*	-.16	-.16	.85***	**.73**					
8. ASDS re-experiencing	1.84 (0.68)	.44***	.43***	.18	-.05	-.20*	.85***	.61***	**.68**				
9. ASDS avoidance	1.67 (0.65)	.13	.18	.05	-.07	-.21*	.72***	.48***	.53***	**.61**			
10. ASDS arousal	1.84 (0.68)	.37**	.38**	.23	-.20	-.24**	.90***	.62***	.73***	.60***	**.77**		
11. Operational Experience	37.40 (37.88)	-.04	-.04	.14	.23	.24**	-.13	-.15	-.15	-.01	-.08	—	
12. Perceived control	3.62 (0.94)	-.01	-.25	-.12	.17	-.05	-.19*	-.17	-.16	-.12	-.17	-.00	—
13. Perceived coping	4.22 (0.74)	-.12	-.13	-.10	.18	-.07	-.06	-.11	-.03	-.02	-.03	-.10	.54***

HSCL-25, Hopkins symptom checklist; GHQ-12, General health questionnaire; ASDS, Acute Stress Disorder Scale. Reliability coefficients (Cronbach’s alpha) are presented in bold on the diagonal.

a Correlation between the two items of the scale.

b Proportion of males in the sample.

*p < .05; **p < .01; ***p < .001.

### Symptom severity and distribution

The overall ASDS score in our sample was *M* = 1.91 (*SD* = 0.60). Applying the criteria developed by Bryant and colleagues (41) to our T2 sample (n=118) yielded a total of 33 (27.97%) probable cases of clinically significant symptoms of ASD. The 95% confidence interval for this prevalence, calculated using the Wilson method, ranged from 20.7% to 36.7%.

### Pre-exposure and peritraumatic predictors of acute stress

The ASDS scores exhibited some signs of non-normality, with skewness ranging from 0.94 to 1.28 and kurtosis between 3.3 and 4.5 across the total and subscale scores. Residual diagnostics further indicated that the Shapiro-Wilk test was statistically significant in all five regression models (*p* <.05), suggesting a violation of the normality assumption. This was visually supported by Q-Q plots and histograms, although the deviations from normality appeared modest. Notably, the normality assumption is generally considered the least critical among regression assumptions and tends to affect hypothesis testing primarily in very small samples (e.g., N ≈ 10), particularly when other assumptions are met and no substantial outliers are present (45). In our case, post-regression diagnostics revealed no evidence of heteroskedasticity, multicollinearity, or influential observations, supporting the robustness of our models. Nonetheless, we conducted supplementary analyses using log-transformed ASDS variables. These yielded results that were largely consistent with the original models, with only minor differences in the reported *p*-values.

The results from our analyses predicting total scores on ASDS and the subscales from the pre-exposure variables are presented in **Table 3**. The results show that only the depression and anxiety subscales from HSCL-25 had any statistically significant positive relationships with scores on ASDS. Higher levels of depression were positively associated with ASDS total (*b* = 0.79; β = .32) and the two subscales dissociation (*b* = 1.30; β = .38) and re-experiencing (*b* = 0.88; β = .36), while higher levels of anxiety were positively associated with ASDS total (*b* = 0.98; β = 0.29) and the re-experiencing subscale only (*b* = 1.03; β = .31). Combined, our variables explained 26.7% of the variance in total ASDS (*R* ^2^ = .267), 29.6% of the variance in dissociation (*R* ^2^ = .296), and 25.7% of the variance in re-experiencing (*R* ^2^ = .257).

**Table 3:** Results from multiple regressions regressing symptoms of acute stress disorder on pre-exposure variables (n = 62).

Variables	ASDS total		ASDS dissociation		ASDS re-experiencing		ASDS avoidance		ASDS arousal	
	Estimate (SE)	*p*	Estimate (SE)	*p*	Estimate (SE)	*p*	Estimate (SE)	*p*	Estimate (SE)	*p*
HSCL-25 depression	0.79 (0.39)	.048*	1.30 (0.53)	.017*	0.88 (0.39)	.028*	0.16 (0.50)	.756	0.64 (0.50)	.204
HSCL-25 anxiety	0.98 (0.48)	.046*	1.26 ^a^ (0.65)	.058	1.03^b^ (0.48)	.038*	0.60 (0.61)	.342	0.91 (0.61)	.142
GHQ-12	-0.19 (0.38)	.629	-0.30 (0.52)	.568	-0.38 (0.38)	.319	-0.16 (0.49)	.753	0.02 (0.49)	.975
Self-efficacy	-0.01 (0.74)	.941	0.00 (0.12)	.989	0.08 (0.09)	.348	-0.01 (0.11)	.915	-0.07 (0.11)	.535
*R* ^2^	.267		.296		.257		.034		.182	
*F*	5.18**		6.00***		4.93**		0.50		3.17*	

HSCL, Hopkins symptom checklist; GHQ-12, General health questionnaire 12 item version; ASDS, Acute Stress Disorder Scale.

a The effect of anxiety was statistically significant in the analysis with log-transformed ASDS dissociation (*p* = .046).

b The effect of anxiety was statistically non-significant in the analysis with log-transformed ASDS re-experiencing (*p* = .051).

*p < .05; **p < .01; ***p < .001.

Values presented in bold are statistically significant.

**Table 4** presents the results from the series of regressions involving operational experience, personnel class, sex, and the peritraumatic variables. Post-regression diagnostics again indicated signs of non-normal residuals, prompting supplementary analyses using log-transformed ASDS variables. Any discrepancies in reported *p*-values between the original and transformed models are noted in the table. Because of a missing value on the variable perceived control, our sample was reduced to *n* = 117.

**Table 4:** Results from multiple regressions regressing symptoms of acute stress disorder on operational experience and peritraumatic variables (n =117).

Variables	ASDS total		ASDS dissociation		ASDS re-experiencing		ASDS avoidance		ASDS arousal	
	Estimate (SE)	*p*	Estimate (SE)	*p*	Estimate (SE)	*p*	Estimate (SE)	*p*	Estimate (SE)	*p*
Operational Experience	-0.00 (0.00)	.835	-0.00 (0.00)	.546	-0.00 (0.00)	.719	0.00 (0.00)	.365	0.00 (0.00)	.619
Perceived control	-0.14 (0.07)	.054	-0.14 (0.10)	.143	-0.13 (0.08)	.108	-0.11 (0.08)	.169	-0.15 (0.08)	.060^c^
Perceived coping	0.02 (0.09)	.820	-0.05 (0.12)	.693	0.03 (0.10)	.772	0.05 (0.10)	.645	0.08 (0.10)	.429
Sex: female	0.37 (0.15)	**.017** ^a^	0.31 (0.21)	.149	0.34 (0.17)	.055^b^	0.41 (0.17)	**.020**	0.44 (0.18)	**.013**
Rank: officer	-0.18 (0.16)	.278	-0.22 (0.23)	.331	-0.26 (0.18)	.165	-0.10 (0.18)	.582	-0.13 (0.19)	.507
Rank: other	-0.24 (0.15)	.108	-0.18 (0.20)	.379	-0.40 (0.17)	**.019**	-0.15 (0.17)	.353	-0.23 (0.17)	.183
*R* ^2^	.116		.082		.126		.073		.109	
*F*	2.42		1.64		2.64		1.45		2.25	

The base category for the rank variable is apprentices/conscripts. HSCL, Hopkins symptom checklist; GHQ-12, General health questionnaire 12 item version; ASDS, Acute Stress Disorder Scale.

a The effect of sex was statistically non-significant in the analysis with log-transformed ASDS dissociation (*p* = .132).

b The effect of sex was statistically significant in the analysis with log-transformed ASDS re-experiencing (*p* = .047).

c The effect of perceived control was statistically significant in the analysis with log-transformed ASDS arousal (*p* = .05).

Values presented in bold are statistically significant.

In these analyses, female sex emerged as the most consistent predictor, showing positive and statistically significant associations with the ASDS total score (*b* = 0.37) as well as the avoidance (*b* = 0.41) and arousal (*b* = 0.44) subscales. Additionally, personnel categorized as “other” scored significantly lower on the re-experiencing subscale compared to apprentices and conscripts (*b* = -0.40). Collectively, the explanatory variables accounted for 11.6% of the variance in the ASDS total score (*R*² = .116), and between 7.3% and 12.6% across the subscale scores.

## Discussion

The present study replicates and extends previous research in several ways. The finding that a significant minority of participants (28.8%) were identified as probable cases of clinically significant symptoms of ASD affirms the severity of the accident and supports its classification as a PTE.

### General psychological distress

No significant relationship was found between pre-incident psychological distress as measured by GHQ-12 and any score on ASDS, thus not supporting H1. Low mean scores and lack of variation in scores may have limited the possible predictive value of GHQ-12 in our sample. Descriptive statistics reveal that only seven participants obtained mean scores ≥2 with no participants scoring >2.25. With scores <2 indicating absence of distress or a feeling of higher functioning than normal, it appears that participants were either very well-functioning at the time of screening or possibly underreporting their level of distress.

### Symptoms of anxiety and depression

Higher pre-incident symptom levels of anxiety and depression predicted higher total scores on ASDS, supporting H2 and H3. These findings align with previous research (16, 46) and suggest that pre-incident symptom level could help identify individuals at elevated risk of ASD symptoms.

One might question whether the results merely demonstrate that measures of distress at different timepoints are correlated. However, while HSCL-25 is a measure of general anxiety and depression, ASDS measures specific distress related to a particular event. Over the six-month period between assessments, participants experienced a serious accident and were exposed to numerous other factors that could have influenced ASD symptoms. The statistically significant link between baseline symptoms and ASDS, despite all other factors influencing symptoms of ASD - such as varying degrees of exposure and differing experiences of fear during the accident - demonstrates the potential utility of baseline symptoms as a predictor. Although the study did not control for degree of exposure, it is reasonable to assume that exposure varied randomly among participants and did not align with baseline symptom levels. Our findings therefore indicate that baseline symptoms of anxiety and depression may serve as a predictor of ASD symptoms, even when accounting for differences in exposure, social support, time of service, and other factors.

#### Baseline symptom levels in a military population

Several studies have attempted to establish appropriate cut-off values for HSCL-25, to accurately identify individuals with clinically significant conditions. A mean score ≥ 1.75 has been used as a cut-off in various studies, but specificity and sensitivity in identifying diagnosable conditions have not been satisfying (47). Consequently, it has been suggested that elevated HSCL-25 scores should rather be interpreted “*as an indicator of psychosocial stress rather than a diagnostic condition”* (48). A large study of a Norwegian student population (*n* = 49 321) – similar in age distribution to the military sample in our study – found that 48% of females and 27% of male respondents in a 2018 survey had mean HSCL-25 scores ≥ 1.75 (48).

Mean total score on HSCL-25 for our military sample was *M* = 1.18 (*SE* = 0.02), with 92.5% of participants scoring <1.5 and no participants scoring >1.68. Our military sample thus demonstrates relatively low levels of psychosocial stress compared to a comparable civilian sample. While low scores may be expected in a selected military population, and while underreporting could possibly influence results, there was still a significant relationship between baseline scores on HSCL-25 and symptoms of ASD. A notable finding from our study is therefore that even minor variations in baseline symptom level in a selected and trained military population may predict an elevated risk of severe symptoms of ASD. This finding has potential implications for personnel care following critical incidents and underscores the need for targeted, preventive interventions.

### Implications regarding sex

Sex was a significant predictor of ASD symptoms at T2, with female sex predicting more symptoms, supporting H4. Controlling for symptoms of general distress, anxiety and depression at T1 did not change this finding, as female crew members did not exhibit significantly higher symptom levels at T1. This suggests that sex itself plays and independent role in expected severity of ASD symptoms. Results are in line with previous research regarding the significance of sex on symptom development following PTE’s (25, 49).

The finding implies that practitioners may expect higher ASD symptom levels among female personnel involved in PTEs, and that practitioners could prepare tailor-made measures aimed at supporting female personnel. Handling this expectation in practice may, however, present challenges, as practitioners must balance sensitivity and targeted prevention efforts with the risk of stigmatization or creating self-fulfilling prophecies. For instance, while organizing all-female group sessions as part of a psychosocial intervention might be intended to provide enhanced support, such an approach could also backfire and inadvertently reinforce sex-based stigma or reduce cohesion in the larger group. Effective and inclusive practical solutions to these challenges should be a topic in future discussions of personnel care following PTEs.

### Other findings

Professional self-efficacy measured at T1 was not predictive of scores on ASDS, not supporting H5. This result suggests that personnel with high professional self-efficacy before a PTE appear to be at the same risk of developing significant symptoms of ASD following a PTE. While high self-efficacy is generally associated with greater initiative, perceived control, and positive outcome expectations (39), self-efficacy appears not to protect against development of ASD symptoms. There could be various reasons for this finding. While self-efficacy is generally seen as protective against post-traumatic stress symptoms (PTSS), studies have also found more complex relationships between self-efficacy and PTSS. Soravia and colleagues (50) found self-efficacy to be protective and associated with lower PTSS among psychiatric and emergency nurses, but a risk-factor for police officers, firefighters and ambulance personnel. In discussing this finding, the authors present overestimation of own ability and reduced controllability and predictability of situations as possible explanations, stating that being too confident in one’s own abilities of dealing with a situation might lead to higher psychological strain when confronted with unexpected and uncontrollable situations (50). This line of reasoning could also be appropriate to the findings of our study; some crew members may have been overly confident in their abilities beforehand and may have experienced the accident situation as unexpected and uncontrollable, thus undermining their sense of self-efficacy.

While pre-incident self-efficacy was not found to be protective, perceived control in the situation was significantly associated with ASDS total score and score on the ASDS subscale arousal. This finding indicates that higher degree of experienced control is associated with less symptoms of ASD, thus supporting H6. The finding is in accordance with social-cognitive theory on stress and trauma (28) as well with previous research (51–53).

Perceived coping did not predict any scores on ASDS, not supporting H7. The finding demonstrates that even personnel who perceive a high degree of coping may develop significant symptoms of ASD following a PTE. Results show high mean scores (*M* = 4.22, *SD* = 0.74), indicating that most crew members felt capable of managing the situation, despite the lack of a protective effect on symptom development.

When scrutinizing the wording of the question regarding perceived coping, we recognize that there could potentially be some confusion regarding what is being measured. While intended as a question regarding how crew members handled the accident and the dramatic situation they were in, the wording of the question could be interpreted as referring to how respondents have coped with their own reactions in the time following the accident. Coping with a dramatic situation differs conceptually from coping with ones’ own psychological reactions following the situation. This ambiguity raises concerns about the validity of the measure.

Operational experience was not predictive of scores on ASDS, not supporting H8. This suggests that personnel across various levels of operational experience reported a wide range of ASD symptoms, indicating that prior experience does not necessarily offer protection against severe stress reactions following a PTE.

Regarding H9, addressing differences between personnel categories, results in **Table 4** show a tendency for officers and other ranks to report less symptoms than apprentices and conscripts. Overall, this tendency in results does not reach significance, and we therefore conclude that H9 is not supported.

### Limitations and methodical approach

The current study has several limitations. First, the study is based on data originally collected and used for routine screening and clinical purposes, rather than a planned data collection for research purposes. While using real-world data enhances ecological validity, it simultaneously limits the possible scientific rigor of the study, constrains the possible participants to be recruited, and restricts the hypotheses that can be tested. The number of participants may limit statistical power, and caution is required when interpreting results, particularly regarding sex, as the number of female participants was low (n=13 at T1 and n=18 at T2). Additionally, possible predictors at T1 were restricted to the factors covered in routine pre-deployment screening. Such limitations are not particular to our study but are common in research on real-world critical incidents. However, to advance knowledge we should seize opportunities and accept less-than-perfect research design, while being mindful of limitations inherent in such studies.

Second, our study relies on a self-report questionnaire developed according to DSM-IV criteria to classify symptoms of ASD. As self-report questionnaires have known limitations in correctly identifying cases of a disorder (54) and as the DSM-IV is no longer in use, this obviously limits the timeliness of results and the confidence in our conclusions. Supplementing the self-report measure with a clinician-administered diagnostic instrument would have improved confidence in results.

Third, aside from physical presence onboard at the time of the accident, we have no measure of exposure to the critical incident. Although all participants were present during the dramatic event, there is bound to be variation in the degree of exposure and the different stressors the crew members experienced. Major accidents and their subsequent handling involve the experience of physical danger, but also other stressors such as burden of responsibility and decision-making, perceived helplessness, uncertainty and fear for the safety of fellow crew members, guilt regarding one’s actions or failures, to mention some. The degree of exposure to a PTE is the main etiological factor in the development of ASD, and variation in exposure could therefore be expected to influence symptom load. No measure of exposure was included in the questionnaires used. While a scaled measure of exposure would have been a strength to the study and is something to strive for in future studies, the current data set did not provide opportunities to control the for degree of exposure.

Fourth, our measure of operational experience may be somewhat misleading, as some crew members may have served on other classes, such as corvettes or submarines, while not having served much time on frigates. Hence, their operational experience on naval vessels, and consequently their training level and experience with battle damage repair and handling critical incidents aboard, may be longer than appears in the data.

Fifth, our measure of self-efficacy is based on only two items, and our measures of perceived coping and perceived control are based on only single items. These are narrow measures with limited reliability and construct validity.

Concerning the choice of methodological approach, an alternative approach could have been to identify cases of likely diagnosable ASD based on ASDS scores and then investigate potential antecedents for these cases. However, as the ASDS has not been updated to reflect changes to diagnostic criteria from DSM-IV to DSM-V, ASDS scores cannot accurately determine the incidence of diagnosable cases according to current criteria. We recommend that the ASDS be updated and validated according to DSM-V criteria.

### Implications for future research and practice

Regarding future research, further studies investigating predictors of ASD are needed. Studies similar to those conducted on post-traumatic stress, using algorithms and machine learning to combine biomarkers, demographic data, data from medical records, brief screening and initial assessment data, are warranted. As suggested by Shalev and colleagues (15) regarding PTSD, producing individual likelihood estimates for ASD may be more feasible than attempting to predict individual cases. Military organizations and populations could serve as appropriate venues for such research.

An important factor in the development of ASD is the degree of exposure to the stressor(s) of a PTE. Measuring degree of exposure can be approached in terms of hard data, such as physical proximity and duration, but exposure is also largely a matter of subjective experience, involving factors such as perception of threat, experienced fear, and emotional proximity. To assess both objective and subjective aspects of exposure, future studies could benefit from a mixed-methods approach.

On the practical side, results can have implications for preventive efforts following PTEs, as they demonstrate that even slightly elevated pre-incident symptoms of anxiety and depression increase the likelihood of developing more severe symptoms of ASD. Even in selected populations some baseline prevalence of minor psychological symptoms is to be expected, and it is not feasible to offer treatment or exclude all personnel experiencing some psychological symptoms. Pre-deployment screening or yearly routine screening could provide data with potential to function as a guide for more targeted prevention following PTEs. Results also show female personnel being at higher risk for severe symptoms compared to male personnel. This implies particular consideration of females in indicated prevention efforts, while also requiring a cautious approach to avoid stigma and self-fulfilling prophecies. Engaging female operational personnel in discussions and development of preventive measures and approaches could be a way to move forward.

Results from current and future research should be incorporated into organizations’ routine screening instruments and personnel care routines following PTEs. Combining measures with predictive value should increase precision in targeting subgroups in indicated preventive efforts. The content of such preventive efforts is another area in need of research; empirical studies are needed to document what preventive efforts are useful for those at high risk of ASD. In the current study, we do not know the impact of the preventive psychosocial intervention on symptom occurrence.

The presented results pertain to a sample of selected and trained military personnel, all of whom participated in a comprehensive psychosocial intervention with a stated intention of preventing acute and post-traumatic stress. Findings indicate that despite comprehensive preventive efforts and high satisfaction with procedures for personnel care and the mentioned intervention (6), significant symptoms of ASD are still to be expected in a minority of participants. Considering these results, an important question concerns the practical significance of such ASD symptom levels to continued service and later operational readiness. As described by Sanden and colleagues (6), the crew in our study was kept intact, received professional support and continued to serve and sail together. Crew members with high levels of ASD symptoms received support from mental health practitioners as needed but were not excluded from the crews’ activities in any way. Despite over a quarter of participants reporting significant symptom levels of ASD three weeks after the accident, later operational readiness was maintained and crew members with previously high levels of ASD symptoms were also performing to standards. This demonstrates how initial symptoms following PTEs can be managed and need not affect future operational readiness of a unit exposed to a PTE.

## Conclusion

Predicting increased risk for ASD is important for enhancing early preventive efforts following PTEs. The current study demonstrates how data from pre-deployment routine screening may help identify subgroups at elevated risk for developing more severe symptoms of ASD. Future research should explore the potential of augmented routine pre-deployment screening, investigating combinations of historical medical data, biomarkers and self-report instruments. Additionally, possibilities for acute screening following a PTE should be further examined, particularly the effectiveness of biomarkers and short screening instruments in predicting ASD and need for treatment.

## Data Availability

The data analyzed in this study is subject to the following licenses/restrictions: The data is owned by the Norwegian Armed Forces Health Registry. Data are available from the authors with the permission of Norwegian Armed Forces Health Registry. Requests to access these datasets should be directed to the Norwegian Armed Forces Health Registry, e-mail: fsan.fhr.datautlevering@mil.no, further inquiries can be directed to the corresponding author/s.
